# High-Protein Nutritional Supplements Improve Nutritional Status in Malnourished Patients with Systemic Sclerosis

**DOI:** 10.3390/nu16162622

**Published:** 2024-08-09

**Authors:** Anna Wojteczek, Jolanta Dardzińska, Marcin Ziętkiewicz, Żaneta Smoleńska, Zenobia Czuszyńska, Diederik De Cock, Zbigniew Zdrojewski, Sylwia Małgorzewicz, Michał Chmielewski

**Affiliations:** 1 Department of Rheumatology, Clinical Immunology, Geriatrics and Internal Medicine, Medical University of Gdańsk, Dębinki 7, 80-211 Gdańsk, Poland; marcin.zietkiewicz@gumed.edu.pl (M.Z.); zaneta.smolenska@gumed.edu.pl (Ż.S.); zenobia.czuszynska@gumed.edu.pl (Z.C.); zbigniew.zdrojewski@gumed.edu.pl (Z.Z.); michal.chmielewski@gumed.edu.pl (M.C.); 2 Department of Clinical Nutrition, Medical University of Gdańsk, Dębinki 7, 80-211 Gdańsk, Poland; jolanta.dardzinska@gumed.edu.pl (J.D.); sylwiam@gumed.edu.pl (S.M.); 3 Biostatistics and Medical Informatics Research Group, Department of Public Health, Vrije Universiteit Brussel, 1090 Brussels, Belgium; diederik.de.cock@vub.be

**Keywords:** systemic sclerosis, nutritional status, cachexia, pre-cachexia, high-protein nutritional support

## Abstract

(1) Background: Impaired nutritional status in systemic sclerosis (SSc) is prevalent. (2) Objective: This study aimed to identify pre-cachexia and malnutrition in SSc patients and to estimate the effectiveness of a high-protein oral nutritional supplement (ONS) in improving their nutritional status. (3) Materials and methods: The SSc population comprised 56 patients and a control group of 49 healthy persons. After a baseline clinical evaluation, bioelectrical impedance analysis (BIA), and laboratory tests, SSc patients were divided into well-nourished, pre-cachectic, and malnourished categories. SSc patients with a nutritional disbalance received a high-protein ONS once daily for 3 months. Patients were reassessed at 3 and 12 months after inclusion in the study. (4) Results: SSc patients, in comparison to the control group, had a significantly lower seven-point SGA value [6(0) vs. 7(1), *p* < 0.001)], lean tissue mass [LTM, 35.1 (10.5) vs. 40.1 (10.10), *p* = 0.008], and lean tissue index [LTI, 13.5 (3) vs. 14.9 (4), *p* = 0.009]. Of the 56 SSc patients, 40 (71.4%) were well nourished, 5 (8.9%) had pre-cachexia, and 11 (19.7%) were malnourished. A high-protein ONS in the pre-cachexia group stabilized the SGA value, anthropometric measurements, and BIA after 3 and 12 months. In malnourished patients, it significantly improved the SGA value [5(0) vs. 6(0), *p* = 0.002], LTI [12.1 (2.1) vs. 12.7 (3.2), *p* = 0.021] and LTM [31.1 (7.7) vs. 35.1 (9.1), *p* = 0.021], and that effect remained stable at 12 months. (5) Conclusion: Malnutrition is a common complication of SSc that can be improved with nutritional intervention.

## 1. Introduction

Systemic sclerosis (SSc) is a connective tissue disease where fibrosis and vascular damage induced by immunological processes can lead to considerable health impairment. Complications of this rare condition often affect the gastrointestinal (GI) tract, leading to malnutrition in patients suffering from SSc [1]. It may even affect over half of SSc patients, depending on the method used to assess its presence [2]. Malnutrition is a serious clinical challenge as it reduces patients’ quality of life and functional status, increases the risk of infectious complications, and prolongs hospitalization time [3,4]. Moreover, it is a well-acknowledged independent risk factor for mortality [5]. Therefore, it is essential to identify risk factors, recognize the early stages of nutritional disbalance, and implement appropriate dietary support to improve patients’ prognosis, general condition, and clinical response to treatment. Many elements can influence nutritional status, such as inflammation, appetite, physical and mental functioning, and the absorption of nutrients [3]. Increased risk of developing malnutrition in the course of SSc stems from disease severity, diffuse cutaneous involvement, and GI symptoms, such as poor appetite, early satiety, nausea, constipation, and diarrhea [6]. The high prevalence of malnutrition in patients with severe and active SSc remains a clinical challenge [7]. SSc patients with severe GI involvement, usually with small intestinal dysmotility, sometimes require feeding via a tube (nasoenteral or per stomal tubes) or even parenteral nutrition when other supportive ways of nourishment are ineffective [8].

However, one of the options used in undernutrition treatment, with proven effects in chronic diseases, is a high-protein oral nutritional supplement (ONS) [9]. Multiple interventional studies with a high-protein ONS strategy have confirmed its efficacy in improving indices of nutrition, and reducing mortality and rehospitalizations [10,11,12,13]. The benefits of a high-protein ONS might be particularly important in the early stages of malnutrition (pre-cachexia), where treatment is thought to be cheaper, safer, and probably more effective than in patients with established malnutrition [14].

In this study, we assessed the difference in nutritional status between patients with SSc and healthy controls. Secondly, we aimed to examine the effects of a 3-month treatment with a high-protein ONS in SSc patients with nutritional disbalance.

## 2. Materials and Methods

This prospective study recruited patients with SSc in the period between 2013 and 2019 in a large university-based hospital. All patients with SSc met the 2013 American College of Rheumatology (ACR)/European League Against Rheumatology (EULAR) classification criteria. Exclusion criteria were age < 18 years and acute infection. A control population was recruited that included healthy volunteers in whom chronic diseases with proven effects on nutritional status had been excluded. The control group was age and sex matched. In both groups, the diet was typical of Middle European cuisine, rich in meat, especially pork and chicken, in addition to a wide range of vegetables, spices, eggs, cereals, and groats.

After these baseline assessments, SSc patients were divided into well-nourished, pre-cachectic, and malnourished groups. The SSc patients with diagnosed pre-cachexia and malnutrition remained in the study and received dietary recommendations of 200 mL of an ONS containing 250 kcal and 19 g of protein (Nestle Resource Protein^®^). The frequency of ONS intake was once a day. A high-protein ONS strategy was continued for 3 months. At 3 and 12 months after baseline, the SSc patients had follow-up visits, during which the same clinical assessment was performed as at baseline.

At the time of study initiation, the following assessments were performed on every patient with SSc and those in the control group.

### 2.1. Clinical Assessment

Clinical evaluation in SSc patients consisted of an assessment of the thickening of the skin and its extension in the modified Rodnan skin score (mRSS), a subtype of SSc (diffuse cutaneous dcSSc or limited cutaneous lcSSc), disease duration (defined from the time of the first symptom meeting ACR/EULAR 2013 criteria with the omission of Raynaud’s phenomenon), and the disease severity scale (DSS) with an assessment of nine systems such as general, peripheral vascular, skin, joint/tendon, muscles, GI tract, lungs, heart, and kidneys. In the DSS, each system was scored from 0 (no involvement) to 4 (end-stage disease). The DSS is the scale created by Medsger et al. [15].

### 2.2. Anthropometric Measurements, Body Composition, Weight Loss, and Appetite

Anthropometric measurements consisted of weight (kg), height (m), body mass index (BMI) calculated from formula kg/m^2^, handgrip strength (HGS) evaluated with MDS Baseline Smedley (kg), triceps skinfold thickness (TSF) measured with a Holtain Skinfold Caliper (mm), and circumferences (cm) of mid-arm (MAC), waist (WC), and hip (HC). Electrical multi-frequency bioimpedance analysis (BIA) was used to evaluate body composition (BCM, Fresenius SA). Each participant was asked about unintentional weight loss of usual body weight during the last 6 months. Appetite was assessed with a Simplified Nutritional Appetite Questionnaire (SNAQ) with a recommended cutoff of ≤14, indicative of anorexia and inadequate dietary intake, and risk of body weight loss in the next 6 months [16]. SNAQ is a valid, efficient, and reliable tool derived from studies based on older adults with chronic comorbidities [17,18].

### 2.3. Laboratory Tests

Routine laboratory tests were performed, such as a complete blood count (CBC), erythrocyte sedimentation rate (ESR), C reactive protein (CRP), prealbumin, albumin, vitamin B1, total cholesterol, high-density lipoprotein cholesterol (HDL), low-density lipoprotein cholesterol (LDL), and triglycerides (TG). In addition, more-sensitive inflammatory parameters like interleukin 6 (IL-6) and high-sensitivity C-reactive protein (hsCRP) were evaluated.

After these baseline assessments, SSc patients were divided into well-nourished, pre-cachectic, and malnourished groups.

Diagnosis of pre-cachexia was based on the following:

(1) The presence of chronic disease
(2) Unintentional weight loss < 5% of usual body weight during the last 6 months
(3) Chronic inflammation identified through increased CRP or ESR
(4) Anorexia or anorexia-related symptoms [13].

Diagnosis of malnutrition was based on a 7-point subjective global assessment (7-point SGA) and serum albumin concentration. The 7-point SGA consists of two main domains: medical history and physical examination. Changes in body weight over the past 6 months, dietary intake, gastrointestinal symptoms, and functional capacity were obtained through an interview. Physical examination focused on the presence of edema and visual and palpatory assessment of subcutaneous fat and muscle tissue. Points 1–2 indicated severe malnutrition, 3–5 indicated mild/moderate malnutrition, and 6–7 indicated a good nutritional status.

The diagnosis of malnutrition was made when the following were met:

(1) The 7-point SGA score was between 1 and 5

or

(2) The serum albumin concentration was lower than <34 g/L [19].

Only SSc patients with diagnosed pre-cachexia or malnutrition were eligible for a dietary intervention with 200 mL once a day of an ONS containing 250 kcal and 19 g of protein. This high-protein ONS strategy was continued for 3 months. At 3 and 12 months after inclusion into the study, the treated SSc patients had a follow-up visit, during which the same clinical assessment was performed as at baseline.

### 2.4. Statistical Analysis

Statistical analyses were performed using IBM SPSS Statistics 26.0. To compare groups in terms of categorical variables, analysis was performed with the χ2 test (when the expected number was greater than 5) or Fisher’s exact test (when the expected number was less than 5). Mann–Whitney U or Kruskal–Wallis tests were used to compare groups in terms of continuous variables, assuming a non-parametric distribution of the data. When two paired measurements were compared, the Wilcoxon signed-rank test was used, and when there were more than 2 paired measurements, the Friedman test was used. The significance level was set below 0.05. This research project was approved by the independent Ethics Committee of the Medical University of Gdańsk (no. NKBBN/328/2015, date 15 September 2015). All participants gave informed consent.

## 3. Results

A total of 105 participants were included in the study. The SSc population comprised 56 patients (47 females, 9 males) aged 22–71 [57.5 (15.5)] years. The control population comprised 49 healthy persons aged 25–86 [57 (14)] years, 42 females and 7 males. Twenty-five patients had diffuse cutaneous systemic sclerosis (dcSSc), and thirty-one had limited cutaneous systemic sclerosis (lcSSc). The mean duration of the disease was 10.6 ± 8.2 years.

In the 56 SSc patients, as compared to the 49 controls, a significantly lower muscle strength [18.33 (9.67) vs. 24 (10.42); *p* < 0.001], a smaller hip circumference [99 (9.25) vs. 103.7 (16.62); *p* = 0.024], and lower seven-point SGA values [6 (0) vs. 7(1); *p* < 0.001] were observed. In the bioimpedance analysis (BIA), a decreased lean tissue mass [35.1 (10.5) vs. 40.1 (10.10); *p* = 0.008] and lean tissue index [13.5 (3) vs. 14.9 (4); *p* = 0.009] were observed in the SSc group. Additionally, the SSc patients demonstrated less total body water, extracellular water, intracellular water, and body cell mass.

The control group had higher serum albumin values [39 (4) vs. (39) 5; *p* = 0.024], hemoglobin [13.9 (1.4) vs. 13.2 (1.5); *p* = 0.007], lymphocytes [2.12 (0.55) vs. 1.725 (0.72); *p* = 0.001], and HDL cholesterol [58 (15) vs. 46 (17.5); *p* = 0.001] in laboratory tests. SSc patients had higher erythrocyte sedimentation rates [15 (17) vs. 8 (10); *p* < 0.001], interleukin-6 [3.16 (5.33) vs. 2.07 (2.58); *p* = 0.001], and triglycerides [121 (83.5) vs. 107 (57.5); *p* = 0.033]. Table 1 shows the baseline comparison between SSc patients and the control group.

Of the 56 patients, 40 (71.4%) were well nourished, 5 (8.9%) had pre-cachexia, and 11 (19.7%) were malnourished. No pre-cachexia or malnutrition was found in the control group. In SSc patients, the disease duration, type of SSc (limited or diffuse), and skin thickening in mRSS did not differ between the groups. However, considering the disease severity scale (DSS), malnourished patients had features of greater damage in organs such as the muscles, heart, and kidneys (Table 2).

In anthropometric measurements, both malnourished and pre-cachectic patients had a significantly lower body mass [68.5 (15.4) vs. 58.0 (30.0) vs. 55.0 (12.0); *p* = 0.003], hip circumference, and appetite measured by SNAQ than well-nourished SSc subjects. A significantly lower BMI and arm circumference were noted in malnourished patients than in well-nourished SSc patients (Table 2).

Bioimpedance analysis revealed a lower fat tissue index and fat mass in malnourished and pre-cachectic patients compared to well-nourished SSc patients (Table 2).

Laboratory tests showed that serum albumin was significantly lower in malnourished and pre-cachectic SSc patients. Pre-cachectic patients also had lower total and LDL cholesterol levels than the other two groups (Table 2).

No statistically significant results were observed between well-nourished, pre-cachectic, and malnourished SS patients in the other morphology parameters, vitamin B1, prealbumin, and inflammatory parameters such as ESR, CRP, hsCRP, and interleukin 6 (Table S1).

There were no clinically significant differences in treatment and gastrointestinal symptoms between well-nourished, pre-cachectic, and malnourished SSc patients (Tables S2 and S3).

**Figure 1:**
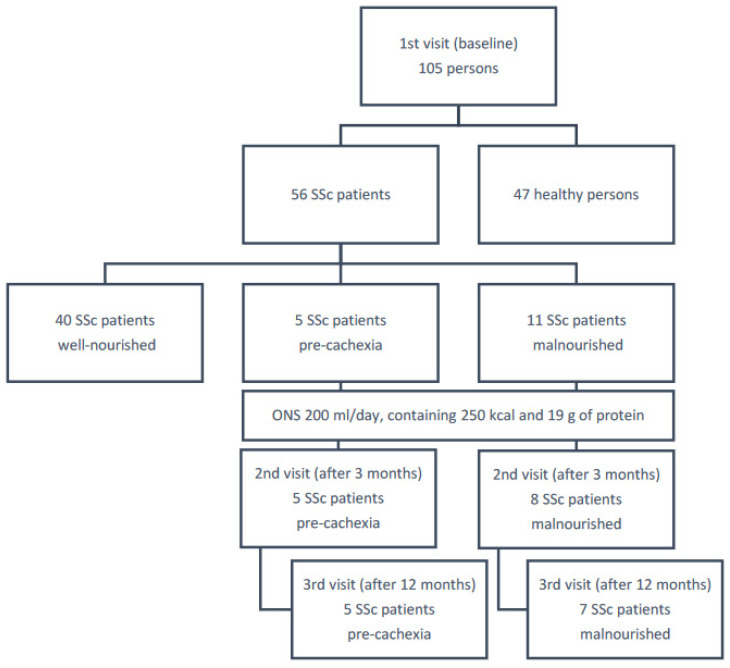
The scheme of the study.

Among all patients with SSc, 16 were diagnosed with nutritional disorders and qualified for further study. Five pre-cachectic and eight malnourished SSc patients (three malnourished persons refused further participation in the study) were given dietary recommendations and a high-protein oral nutritional supplement (200 mL once a day, containing 250 kcal and 19 g of protein) for 3 months and then were re-evaluated for nutrition status. There were no relevant effects on the GI tract of the ONS, and its compliance was without dropouts. Subsequently, 12 months after an initial assessment, seven malnourished subjects (one died because of sepsis) and five subjects from the pre-cachexia group were reassessed as at baseline (Figure 1).

Treatment of pre-cachectic SSc patients with dietary recommendations and a high-protein ONS resulted in stabilization in nutritional status according to the seven-point SGA, appetite measures, anthropometric measurements, and parameters from bioimpedance analysis, both at 3 and 12 months from baseline (Table 3). In laboratory tests, both CRP and hsCRP increased after three months of supplementation, falling back at 12 months. There were no clinically significant differences in other laboratory parameters (Table S4).

In malnourished SSc patients, dietary recommendations and a high-protein ONS significantly improved nutritional status on the seven-point SGA and body composition parameters. At the follow-up visits, the scores for the seven-point SGA, ICW, LTI, LTM, and BCM were significantly higher than at baseline, as shown in Table 4. There were no significant differences in the SGA between the follow-up visits. As for anthropometric measurements, hip and arm circumferences were higher after 12 months than in the previous two measurements. There were no clinically significant differences in other parameters (Table S5).

## 4. Discussion

Systemic sclerosis represents connective tissue diseases in which nutritional imbalance is relatively frequent, and our results confirm the prevalence of this clinically important complication [20]. Furthermore, we showed that a high-protein ONS might be effective in reversing malnutrition in the course of SSc.

There are several potential causes of nutritional disbalance in SSc. Inflammation can be involved, as significantly higher inflammatory parameters were noted in SSc patients than in the controls [21]. The presence of inflammation can also affect sarcopenia, where a generalized loss of muscle mass, strength, and muscle function increases the risk of complications [22]. In the revised criteria of sarcopenia for clinicians, muscle strength and functioning are highlighted. Reduced muscle strength measured with hand grip strength or chair stand test gives a probable diagnosis of sarcopenia and is sufficient for nutritional intervention. Suggested cut-off points in assessing sarcopenia by hand grip strength are <20 kg for women and <30 kg for men [23]. In our population, SSc patients had significantly lower muscle strength compared to the healthy control group. In a previous study by Marighela et al., sarcopenia assessed by relative skeletal mass index (RSMI) evaluated by dual-energy X-ray absorptiometry (DXA) was found in patients with diffuse SSc, compared to the control group. However, there were no differences between the whole SSc population and healthy controls. It was suggested that diffuse SSc was associated with inflammation (higher levels of CRP), which could lead to cachexia and sarcopenia syndromes [24,25]. The inflammatory activity seems to be associated with muscle wasting and may share similar pathways in the pathogenesis of malnutrition [26]. This concept may be indirectly supported by a study by Caimmi et al., where sarcopenia was found to be more prevalent in malnourished compared to well-nourished SSc patients [27]. Moreover, muscle involvement in SSc may not only be the result of sarcopenia. Another important cause of muscle weakness is also myopathy, which is one of the risk factors for severe GI dysmotility, that usually requires enteral or total parenteral nutrition [28].

Myositis, in addition to older age, diffuse cutaneous disease, and opioid use, is one of the proven risk factors for the development of the serious GI complication of pseudo-obstruction [29].

In our study, the laboratory results of the SSc population showed significant differences in inflammatory parameters (ESR and IL-6) and morphology parameters, such as lower total lymphocyte and hemoglobin levels compared to the healthy population. These parameters are valuable markers for determining nutritional status. Levels of total lymphocytes < 0.8 G/L reflect severe malnutrition, and those of <1.5 G/L were proven as a risk factor for higher mortality [30]. However, lower total lymphocyte levels may also result from immunosuppressive treatment, which cannot be ruled out in this study. As for hemoglobin, its lower level has been recognized as a marker of malnutrition, but its concentration depends on many factors, including the acute course of the disease [31]. Moreover, it is one of the parameters typical for DSS, where values below 12.3 g/dL are considered significant in SSc patients [15].

Another essential tool for calculating body composition is electrical multi-frequency bioimpedance analysis (BIA). Differences in body fat measurements, hydration levels, and lean muscle mass were previously noted in patients with SSc, compared to a healthy population. There are several parameters used for assessing the loss of muscle mass. In methods where the body is divided into two compartments, fat-free mass (FFM) is the total body mass except for fat mass (FM). In contrast, lean tissue mass (LTM) is one of the measurements assessed in three body-compartment divisions, besides adipose tissue mass and virtual overhydration [32]. Another parameter that BIA measures, depending on body cell mass and cellular integrity, is the phase angle (PhA) [33]. In our study, SSc patients had significantly lower total body water (TBW), external body water (ECW), internal body water (ICW), body cell mass (BCM), lean tissue mass (LTM), and a lean tissue index (LTI) in comparison with a healthy population. In studies where SSc patients were compared to a healthy population, comparable results were observed. Molfino et al. showed a lower phase angle (PhA), internal body water (ICW), and body cell mass (BCM) values. Similarly, Krause et al. observed a lower phase angle (PhA), body cell mass (BCM), extracellular mass (ECM) values, and an ECM/BCM ratio in the studied SSc groups [2,34].

Growing awareness of the impact of malnutrition on clinical conditions has led to a search for tools to determine factors with an increased risk of eating disorders. In the development of disease-related malnutrition with inflammation (cachexia), the occurrence of early heraldic symptoms such as anorexia and a decrease in body mass of less than 5% has been noted. Such a clinical situation is called pre-cachexia [35]. In our population, the percentage of SSc patients in the pre-cachexic state was relatively high, equaling 8.9%. This seems to be clinically relevant information due to the need for offensive nutritional intervention to prevent malnutrition and its consequences, as confirmed in recent studies [11,36,37].

Several studies were conducted to assess the presence of malnutrition in the SSc population, using various methods for its evaluation. In our study, the prevalence of malnutrition was 19.6% according to the seven-point SGA. The SGA is an established method for measuring malnutrition in many populations and corresponds with the Global Leadership Initiative on Malnutrition (GLIM) criteria, a recent tool evolved to diagnose malnutrition with high sensitivity and specificity [38]. Our results stay in accordance with those of Rosato et al., where the prevalence of malnutrition following the GLIM criteria was 16.6%. However, some studies evaluating malnutrition on the basis of the SGA showed its prevalence to be as high as 50% [39]. These differences in the prevalence of malnutrition, despite using the same assessment method, might have resulted from small samples, and differences in the disease activity and organ involvement of the investigated groups [7].

Risk factors associated with the onset of undernutrition in SSc have been evaluated in several studies. In our study, disease duration and SSc type did not differ between patients with different nutritional statuses, which is comparable to the results obtained by Caporali [40]. However, Baron et al. indicated that a shorter disease duration, probably connected to the dcSSc subtype of the disease, was associated with a higher risk of malnutrition [6]. The same suggestion was made in the Krause study, where patients with higher scores in mRSS had a poor nutritional status [2].

Caporali et. al. observed a relationship between disease activity and undernutrition, but no relationship was found [40]. Different results were obtained in the Rosato study, where malnutrition assessed by the FFMI showed a negative correlation with disease activity and severity [7]. In our study, malnourished patients had more significant involvement of the muscles, heart, and kidneys, as assessed with the nine elements of the DSS. These observations are comparable to the results from Caimmi et al. and confirm the link between the severity of the disease and the occurrence of undernutrition [27]. Moreover, muscle involvement is a proven risk factor for severe GI involvement like pseudo-obstruction requiring nutritional support [28].

According to the GLIM criteria of malnutrition, its most common etiological factors in the SSc population include low BMI, reduced food intake, and a combination of a reduced fat-free mass index (FFMI) and inflammation [7,41]. In our study, low BMI and other anthropometric measurements, such as arm and hip circumference, were significantly lower in malnourished SSc patients than in the well-nourished group. Both pre-cachectic and malnourished patients had a lower appetite, which was proven to be an essential cause of undernutrition in SSc patients. Surprisingly, there was no FFMI, but our population’s fat tissue index and fat mass were lower in malnourished and pre-cachectic patients.

As for laboratory parameters, not surprisingly, the level of serum albumin was lower in malnourished and pre-cachectic SSc patients. Albumin is a valuable biomarker for assessing malnutrition, as demonstrated in a meta-analysis evaluating 43 blood markers [7,40].

When SSc patients were divided into subgroups, the pre-cachexia group had significantly lower total cholesterol and LDL cholesterol. Those results confirm observations in SSc, RA, or SLE populations, where an untreated inflammatory response was associated with lipid-lowering effects [42,43]. Total cholesterol levels lower than 160 mg/dL, when not on lipid-lowering medications, are indicators of malnutrition, and when they drop below 120 mg/dL, mortality increases ten-fold [30].

Several clinical practice guidelines highlight the importance of dietary interventions in nutritional disorders. Those nutritional support strategies include individual dietary counseling, a high-protein oral nutritional supplement (ONS), or artificial nutritional support. In SSc, the demand for protein is increased due to poor appetite, increased protein losses when the digestive tract is involved in SSc, and the catabolic effect of an uncontrolled disease state [10,44].

High-protein ONSs, widely used in acute and chronic diseases, such as hip fractures, cancers, gastrointestinal diseases, chronic ulcers, and chronic obstructive pulmonary disease, effectively reduced mortality, rehospitalizations and wound healing, and improved muscle strength [10].

Previous nutritional interventions conducted in patients with SSc have focused on highly individualized medical nutrition therapy (MNT) based on macro- and micronutrient requirements and proper caloric intake. In the study by Ortiz et al., 9 patients (12.5%) out of 72 with a high risk of malnutrition stratified by the MUST (Malnutrition Universal Screening Tool) were treated with MNT for 12 months. However, no improvements were observed in weight, food intake, and biochemical parameters [45]. In another study where 94 SSc patients with signs of malnutrition were assessed by BIA and received MNT for a mean period of 14.9 months, PhA (phase angle) and PC (percentage of cells) increased. No changes were noticed in BMI, FAT, TBW, LTM, BCM, and ICW [2]. In our study, high-protein ONS with MNT in malnourished SSc patients significantly improved scores in the seven-point SGA, ICW, LTI, LTM, and BCM after 3 months of intervention. This effect persisted after 12 months. In pre-cachectic SSc patients, a high-protein ONS stabilized the seven-point SGA, appetite, BIA parameters, and anthropometric measurements throughout the clinical follow-up. Furthermore, a tendency to increase albumin concentration was observed after 3 months of treatment with a high-protein ONS.

Our study is one of few studies on nutritional support in patients with SSc. It has its limitations. It was a single-center study with a relatively small study group. Moreover, the study group was dominated by patients with lSSc. In addition, only pre-cachectic and malnourished patients were subjected to further clinical observation and evaluation of the impact of a high-protein ONS. In addition, we did not monitor whether diet and exercise remained constant throughout the observation in the undernourished SSc group. Moreover, we conducted numerous statistical tests without adjusting for multiple comparisons, increasing the likelihood of finding false-positive results. However, as this study is exploratory, we did not use an adjusted level of significance. The study confirms the need to evaluate the nutritional status of each SSc patient and then, depending on the result, the need to properly implement nutritional treatment.

## 5. Conclusions

Systemic sclerosis patients are known to be at higher risk of nutritional disbalance. Routine assessment of their nutritional status and implementation of nutritional intervention as early as possible is mandatory. Future research on larger groups should confirm the findings of our study. Moreover, the long-lasting effect of a high-protein ONS should also be assessed in well-nourished SSc patients.

## Figures and Tables

**Table 1:** Baseline comparison between SSc patients and control group. Data are presented as Me—median; IQR—interquartile range.

	Control Group (*n* = 49)	SSc Patients (*n* = 56)	*p*-Value
Sex, *n* (%)			
Women	42 (85.7)	47 (83.9)	0.799
Men	7 (14.3)	9 (16.1)	0.799
Age [y], Me (IQR)	57 (14)	57.5 (15.5)	0.777
SNAQ [points], Me (IQR)	17 (2)	16.5 (3.25)	0.467
SGA [points], Me (IQR)	7 (1)	6 (0)	<0.001
Anthropometric measurements			
Height [cm], Me (IQR)	165 (10)	163.5 (9.75)	0.157
Weight [kg], Me (IQR)	69 (22.25)	67.2 (16.5)	0.070
BMI [kg/m^2^], Me (IQR)	25.7 (7.15)	25.05 (4.45)	0.105
HGS [kg], Me (IQR)	24 (10.42)	18.33 (9.67)	0.001
MAC [cm], Me (IQR)	29 (5.525)	27.75 (4.5)	0.053
WC [cm], Me (IQR)	89 (23.75)	84.5 (16.12)	0.083
HC [cm], Me (IQR)	103.7 (16.62)	99 (9.25)	0.024
WHR, Me (IQR)	0.84 (0.1)	0.86 (015)	0.959
TSF [mm], Me (IQR)	23.87 (10.51)	21.07 (9.55)	0.133
Bioimpedance analysis			
OH [L], Me (IQR)	−0.3 (1.45)	−0.055 (1.62)	0.191
OH [% ECW], Me (IQR)	−2.1 (8.8)	−0.5 (10.9)	0.220
V [L], Me (IQR)	35.1 (9.05)	31.3 (5.8)	0.002
TBW [L], Me (IQR)	36.3 (9.3)	32.65 (6.77)	0.020
ECW [L], Me (IQR)	15.8 (4.25)	14.3 (3.47)	0.044
ICW [L], Me (IQR)	19.5 (3.85)	17.25 (4.22)	0.001
E/I ratio, Me (IQR)	0.8 (0.095)	0.81 (0.14)	0.365
LTI [kg/m^2^], Me (IQR)	14.9 (4)	13.5 (3)	0.009
FTI [kg/m^2^], Me (IQR)	11.7 (7.5)	11.2 (6.1)	0.752
LTM [kg], Me (IQR)	40.1 (10.10)	35.1 (10.5)	0.008
LTM [%], Me (IQR)	58.9 (20.05)	54.8 (16)	0.486
FAT [kg], Me (IQR)	21.9 (16.4)	21.5 (11.4)	0.569
FAT [%], Me (IQR)	29.5 (15.05)	31.8 (12.1)	0.623
ATM [kg], Me (IQR)	29.8 (22.35)	29.3 (15.4)	0.569
BCM [kg], Me (IQR)	22.7 (7.1)	20 (7.3)	0.006
Laboratory measurements			
Hb [g/dL], Me (IQR)	13.9 (1.4)	13.2 (1.5)	0.007
Plt [G/L], Me (IQR)	257 (49.5)	249.5 (92)	0.775
Wbc [G/L], Me (IQR)	5.83 (1.31)	6.65 (3.135)	0.054
Neut [G/L], Me (IQR)	2.93 (1.12)	3.855 (2.20)	0.002
Lymph [G/L], Me (IQR)	2.12 (0.55)	1.725 (0.72)	0.001
Mono [G/L], Me (IQR)	0.51 (0.19)	0.555 (0.29)	0.063
Eos [G/L], Me (IQR)	0.16 (0.12)	0.12 (0.13)	0.050
Baso [G/L], Me (IQR)	0.03 (0.02)	0.02 (0.02)	<0.001
TC [mg/dL], Me (IQR)	216 (54)	211.5 (67.5)	0.321
TG [mg/dL], Me (IQR)	107 (57.5)	121 (83.5)	0.033
HDL [mg/dL], Me (IQR)	58 (15)	46 (17.5)	0.001
LDL [mg/dL], Me (IQR)	141 (48.5)	135 (50)	0.367
ESR [mm/h], Me (IQR)	8 (10)	15 (17)	<0.001
CRP [mg/L], Me (IQR)	1.51 (2.74)	1.74 (3.69)	0.297
hsCRP [mg/L], Me (IQR)	3.84 (5.97)	5.03 (10.06)	0.311
IL-6 [pg/mL], Me (IQR)	2.07 (2.58)	3.16 (5.33)	0.001
Albumins [g/L], Me (IQR)	39 (4)	39 (5)	0.024

Me—median; IQR—interquartile range; SNAQ—Simplified Nutritional Appetite Questionnaire; SGA—subjective global assessment; OH—overhydration; V—volume; TBW—total body water; ECW—extracellular water; ICW—intracellular water; E/I ratio—extracellular-to-intracellular fluid volume ratio; BMI—body mass index; LTI—lean tissue index; FTI—fat tissue index; LTM—lean tissue mass; FAT—fat mass; ATM—adipose tissue mass; BCM—body cell mass; HGS—handgrip strength; MAC—mid-arm circumference; HC—hip circumference; WC—waist circumference; WHR—waist–hip ratio; TSF—triceps skinfold thickness; Hb—hemoglobin; Plt—platelets; Wbc—white blood cells; Neut—neutrophils; Lymph—lymphocytes; Mono—monocytes; Eos—eosinophils; Baso—basophils; TC—total cholesterol; TG—triglycerides; HDL—high-density lipoprotein cholesterol; LDL—low-density lipoprotein cholesterol; ESR—erythrocyte sedimentation rate; CRP—C reactive protein; hsCRP—high-sensitivity C-reactive protein; IL-6—interleukin 6.

**Table 2:** Comparison between well-nourished, pre-cachectic, and malnourished SSc patients. Data are presented as Me—median; IQR—interquartile range.

	Well Nourished (*n* = 40)	Pre-Cachexia (*n* = 5)	Malnourished (*n* = 11)	*p*-Value
Disease duration [y], Me (IQR)	10.5 (14.25)	9 (13.5)	6 (6)	0.272
lcSSc, *n* (%)	24 (60.0)	4 (80.0)	5 (45.5)	0.404
dcSSc, *n* (%)	16 (40.0)	1 (20.0)	6 (54.5)	0.404
mRSS [points], Me (IQR)	6 (6)	5 (4.5)	6 (13)	0.787
DSS skin [points], Me (IQR)	1 (0)	1 (0)	1 (1)	0.257
DSS joint/tendon [points], Me (IQR)	1 (1)	1 (2.5)	1 (2)	0.489
DSS muscle [points], Me (IQR)	0 (0)	0 (0.5)	1 (1)	<0.001
DSS GI tract [points], Me (IQR)	0 (1)	0 (1)	1 (1)	0.120
DSS lung [points], Me (IQR)	1 (2)	0 (1)	2 (2)	0.095
DSS heart [points], Me (IQR)	0 (0)	0 (0)	0 (1)	0.008
DSS kidney [points], Me (IQR)	0 (0)	0 (0)	0 (0)	0.016
DSS in total [points], Me (IQR)	5 (4)	4 (5)	9 (8)	0.009
SNAQ [points], Me (IQR)	17 (3)	13 (2)	13.5 (4.5)	0.006
SGA [points], Me (IQR)	6 (0)	6 (0.5)	5 (1)	<0.001
Anthropometric measurements				
Weight [kg], Me (IQR)	68.5 (15.4)	58 (30)	55 (12)	0.003
BMI [kg/m^2^], Me (IQR)	25.5 (3.82)	24.8 (11.15)	20.7 (5.8)	0.001
MAC [cm], Me (IQR)	28 (3.75)	29.5 (7.75)	24 (7.3)	0.004
HC [cm], Me (IQR)	99.75 (6.62)	96 (20.5)	94 (19)	0.033
Bioimpedance analysis				
LTI [kg/m^2^], Me (IQR)	14.3 (4.275)	13.45 (2.1)	12.2 (2.6)	0.091
FTI [kg/m^2^], Me (IQR)	11.25 (5.25)	9.7 (7.25)	6.1 (7.3)	0.036
LTM [kg], Me (IQR)	37 (12.25)	34.95 (9.4)	33.3 (8.8)	0.235
LTM [%], Me (IQR)	54.5 (16.225)	58.05 (11.925)	64.5 (22.1)	0.278
Fat [kg], Me (IQR)	23 (9.525)	17.6 (15.3)	12.2 (11.8)	0.013
Fat [%], Me (IQR)	33.2 (11.87)	31.05 (10.5)	23 (19.3)	0.185
ATM [kg], Me (IQR)	31.15 (12.97)	23.95 (20.82)	16.6 (16)	0.013
Laboratory measurements				
Hb [g/dL], Me (IQR)	13.4 (1.65)	12.4 (2.95)	12.8 (2.4)	0.086
Plt [G/L], Me (IQR)	248 (92)	245 (51)	277 (178)	0.195
Wbc [G/L], Me (IQR)	6.58 (2.62)	6.81 (7.39)	6.91 (7.13)	0.362
Lymph [G/L], Me (IQR)	1.675 (0.67)	1.97 (1.255)	1.75 (0.9)	0.529
TC [mg/dL], Me (IQR)	218.5 (52.25)	162 (47)	203 (122)	0.043
TG [mg/dL], Me (IQR)	126.5 (82)	113 (54)	112 (123)	0.875
HDL [mg/dL], Me (IQR)	47.5 (16.75)	45 (23.5)	40 (23)	0.220
LDL [mg/dL], Me (IQR)	138 (41)	98 (36)	141 (73)	0.031
ESR [mm/h], Me (IQR)	15 (15)	17 (18.5)	23 (37)	0.461
CRP [mg/L], Me (IQR)	1.52 (3.57)	1.89 (2.47)	2.59 (31.97)	0.151
hsCRP [mg/L], Me (IQR)	4.455 (9.49)	6.32 (9.51)	8.715 (11.43)	0.360
IL-6 [pg/mL], Me (IQR)	3.24 (3.51)	3.16 (6.63)	5.22 (27.29)	0.455
Albumin [g/L], Me (IQR)	39 (4)	33 (9)	35 (8)	0.014

Me—median; IQR—interquartile range; lcSSc—limited cutaneous subset systemic sclerosis; dcSSc—diffuse cutaneous subset systemic sclerosis; mRSS—modified Rodnan skin score; DSS—disease severity scale; SNAQ—Simplified Nutritional Appetite Questionnaire; SGA—subjective global assessment; BMI—body mass index; LTI—lean tissue index; FTI—fat tissue index; LTM—lean tissue mass; FAT—fat mass; ATM—adipose tissue mass; MAC—mid-arm circumference; HC—hip circumference; Hb—hemoglobin; Plt—platelets; Wbc—white blood cells; Lymph—lymphocytes; TC—total cholesterol; TG—triglycerides; HDL—high-density lipoprotein cholesterol; LDL—low-density lipoprotein cholesterol; ESR—erythrocyte sedimentation rate; CRP—C reactive protein; hsCRP—high-sensitivity C-reactive protein; IL-6—interleukin 6.

**Table 3:** The comparison between baseline (visit 1), 3 months after high-protein oral nutritional supplement (visit 2), and 12 months (visit 3) since baseline in SSc patients with pre-cachexia. Data are presented as Me—median; IQR—interquartile range.

	Visit 1	Visit 2	Visit 3	*p*-Value
SNAQ [points], Me (IQR)	13 (2)	14 (3.5)	16 (4)	0.441
SGA [points], Me (IQR)	6 (0.5)	6 (0.5)	6 (0.5)	1.000
Anthropometric measurements				
Weight [kg], Me (IQR)	58 (30)	60.5 (27.7)	62 (22.7)	0.504
MAC [cm], Me (IQR)	29.5 (7.8)	28 (9)	29.5 (6.8)	0.291
HC [cm], Me (IQR)	96 (20.5)	96 (23.3)	102.5 (18.5)	0.838
Bioimpedance analysis				
ECW [L], Me (IQR)	14.2 (4.9)	14.8 (5.8)	15.9 (3.9)	0.091
ICW [L], Me (IQR)	16.5 (15.2)	17.5 (5.9)	18.7 (4.5)	0.819
LTI [kg/m^2^],, Me (IQR)	13.5 (2.1)	14.2 (6.7)	15 (4.6)	0.627
FTI [kg/m^2^], Me (IQR)	9.7 (7.3)	13.3 (11.3)	12.2 (12.2)	0.472
LTM [kg], Me (IQR)	35 (9.4)	37.3 (18.8)	37.2 (14.2)	0.779
LTM [%], Me (IQR)	58.1 (11.9)	56.9 (34.3)	53.4 (33)	0.779
BCM [kg], Me (IQR)	19 (5.5)	21 (13.4)	21.5 (9.8)	0.627
Laboratory measurements				
Hb [g/dL], Me (IQR)	12.4 (3)	12.8 (2.4)	12.4 (1.8)	0.291
Plt [G/L], Me (IQR)	245 (51)	252 (97)	261 (130)	0.041
Wbc [G/L], Me (IQR)	6.8 (7.4)	6.9 (4.6)	7.2 (6.8)	0.549
Lymph [G/L], Me (IQR)	2 (1.3)	2 (1.2)	2.4 (1.7)	0.449
ESR [mm/h], Me (IQR)	17 (18.5)	17 (34)	23 (35.5)	0.257
CRP [mg/L], Me (IQR)	1.9 (2.5)	3.2 (9.3)	2 (3.2)	0.041
hsCRP [mg/L], Me (IQR)	6.3 (9.5)	11.3 (10.4)	2.7 (5)	0.022
IL-6 [pg/mL], Me (IQR)	3.2 (6.6)	5.5 (10.9)	4.9 (6.9)	1.000
Albumin [g/L], Me (IQR)	33 (9)	39 (7)	38 (4.5)	0.056

Me—median; IQR—interquartile range; SNAQ—Simplified Nutritional Appetite Questionnaire; SGA—subjective global assessment; ECW—extracellular water; ICW—intracellular water; BMI—body mass index; LTI—lean tissue index; FTI—fat tissue index; LTM—lean tissue mass; BCM—body cell mass; MAC—mid-arm circumference; HC—hip circumference; Hb—hemoglobin; Plt—platelets; Wbc—white blood cells; Lymph—lymphocytes; ESR—erythrocyte sedimentation rate; CRP—C reactive protein; hsCRP—high-sensitivity C-reactive protein; IL-6—interleukin 6.

**Table 4:** The comparison between baseline (visit 1), 3 months after high-protein oral nutritional supplement (visit 2), and 12 months (visit 3) since baseline in SSc patients with malnutrition. Data are presented as Me—median; IQR—interquartile range.

	Visit 1	Visit 2	Visit 3	*p*-Value
SNAQ [points], Me (IQR)	15 (0)	16 (2)	15 (7)	0.454
SGA [points], Me (IQR)	5 (0)	6 (0)	6 (0)	0.002
Anthropometric measurements				
Weight [kg], Me (IQR)	55 (7)	56.2 (4.7)	55.5 (9)	0.756
MAC [cm], Me (IQR)	24 (5.3)	23.5 (3)	26 (6.5)	0.048
HC [cm], Me (IQR)	95.5 (15)	96.5 (9)	100 (8)	0.008
Bioimpedance analysis				
ECW [L], Me (IQR)	13.1 (3.1)	13.5 (2.1)	14.3 (2.3)	0.008
ICW [L], Me (IQR)	15.3 (2)	16.3 (2.7)	17.6 (2.1)	0.021
LTI [kg/m^2^], Me (IQR)	12.1 (2.1)	12.7 (3.2)	14.4 (4)	0.021
FTI [kg/m^2^], Me (IQR)	9.7 (6.8)	6.3 (6.3)	9.2 (10.2)	0.867
LTM [kg], Me (IQR)	31.1 (7.7)	35.1 (9.1)	36.6 (10.2)	0.021
LTM [%], Me (IQR)	54.1 (18.7)	67.6 (18.2)	61 (36.5)	0.156
BCM [kg], Me (IQR)	16.7 (5)	19.6 (7.3)	20.9 (7.3)	0.021
Laboratory measurements				
Hb [g/dL], Me (IQR)	13.2 (0.7)	13 (0.7)	13.6 (3.1)	0.738
Plt [G/L], Me (IQR)	245 (134)	265.5 (134)	251 (73)	0.846
Wbc [G/L], Me (IQR)	6.8 (10)	7.3 (9.3)	5.6 (2.4)	0.607
Lymph [G/L], Me (IQR)	1.8 (0.5)	2 (1.4)	1.4 (1)	0.311
ESR [mm/h], Me (IQR)	23 (24)	16 (21)	24 (17)	0.756
CRP [mg/L], Me (IQR)	2 (2.4)	3.3 (6.5)	2.7 (2.1)	0.156
hsCRP [mg/L], Me (IQR)	5 (10.4)	8.1 (11.1)	4.6 (3.3)	0.368
IL-6 [pg/mL], Me (IQR)	2.5 (5.3)	2.9 (16.2)	1 (4.5)	0.156
Albumin [g/L], Me (IQR)	39 (5)	36 (4)	39 (3)	0.368

Me—median; IQR—interquartile range; SNAQ—Simplified Nutritional Appetite Questionnaire; SGA—subjective global assessment; ECW—extracellular water; ICW—intracellular water; LTI—lean tissue index; FTI—fat tissue index; LTM—lean tissue mass; MAC—mid-arm circumference; HC—hip circumference; Hb—hemoglobin; Plt—platelets; Wbc—white blood cells; Lymph—lymphocytes; ESR—erythrocyte sedimentation rate; CRP—C reactive protein; hsCRP—high sensitivity C-reactive protein; IL-6—interleukin 6.

## Data Availability

The original contributions presented in the study are included in the article/Supplementary Materials, further inquiries can be directed to the corresponding author/s.

## Supplementary Materials

The following supporting information can be downloaded at: https://www.mdpi.com/article/10.3390/nu16162622/s1, Table S1: Comparison between well-nourished, pre-cachectic, and malnourished SSc patients; Table S2: Comparison in GI symptoms at baseline between well-nourished, pre-cachectic, and malnourished SSc patients; Table S3: Comparison in treatment at baseline between well-nourished, pre-cachectic, and malnourished SSc patients; Table S4: The comparison between baseline (visit 1), 3 months after high protein oral nutritional supplement (visit 2), and 12 months (visit 3) since baseline in SSc patients with pre-cachexia; Table S5: The comparison between baseline (visit 1), 3 months after high protein oral nutritional supplement (visit 2), and 12 months (visit 3) since baseline in SSc patients with malnutrition.
