# Supplementary material for: High-Protein Nutritional Supplements Improve Nutritional Status in Malnourished Patients with Systemic Sclerosis
Source: Nutrients. 2024 Aug 9;16(16):2622. doi: 10.3390/nu16162622 (PMC11357555; doi:10.3390/nu16162622)
Supplement: Supplementary file 1 [file nutrients-16-02622-s001.zip › nutrients-3119694-supplementary.pdf]

**Table S1.** Comparison between well-nourished, pre-cachectic, and malnourished SSc patients. Data are presented as Me – median; IQR – interquartile range.

| | **Well-nourished (n = 40)** | **Pre-cachexia (n = 5)** | **Malnourished (n = 11)** | **pValue** |
|---|---|---|---|---|
| Sex, *n (%)* | | | | |
| Women | 33 (82.5) | 5 (100.0) | 9 (81.8) | 0.861 |
| Men | 7 (17.5) | 0 | 2 (18.2) | 0.861 |
| Age [y], *Me (IQR)* | 57 (15.5) | 61 (27) | 57 (14) | 0.833 |
| AntiScl70-Ab positive, *n (%)* | 25 (62.5) | 1 (20) | 5 (45.5) | 0.133 |
| AntiACA-Ab positive, *n (%)* | 7 (17.5) | 3 (60.0) | 2 (18.2) | 0.154 |
| AntiRNAPIII-Ab positive, *n (%)* | 3 (7.5) | 1 (20.0) | 2 (18.2) | 0.278 |
| unintentional weight loss >6kg of usual body weight during the last 6 months [3 points], n(%) | 1 (2.5) | 0 | 6 (54.5) | **<0.001** |
| **Anthropometric measurements** | | | | |
| Height [cm], *Me (IQR)* | 163.5 (9.5) | 162 (15) | 164 (10) | 0.937 |
| HGS [kg], *Me (IQR)* | 19.33 (10.67) | 16.67 (14.835) | 15.5 (9) | 0.307 |
| WC [cm], *Me (IQR)* | 87 (19.37) | 84 (17.5) | 79 (12.5) | 0.058 |
| WHR [ratio], *Me (IQR)* | 0.865 (0.17) | 0.89 (0.105) | 0.82 (0.16) | 0.538 |
| TSF [mm], *Me (IQR)* | 21.785 (10.80) | 21.07 (13.05) | 16.95 (11.20) | 0.149 |
| **Bioimpedance analysis** | | | | |
| OH [L], *Me (IQR)* | -0.1 (1.3) | -1.5 (7) | 0.5 (4.4) | 0.066 |
| OH [% ECW], *Me (IQR)* | -0.55 (8.97) | -11.6 (43.55) | 5.3 (29) | 0.059 |
| V [L], *Me (IQR)* | 32.6 (6.47) | 29.2 (20) | 29.6 (3.8) | **0.030** |
| TBW [L], *Me (IQR)* | 33.45 (8.38) | 30.6 (20) | 30.5 (9.5) | 0.310 |
| ECW [L], *Me (IQR)* | 14.35 (4.2) | 14.2 (4.85) | 13.1 (4.3) | 0.150 |
| ICW [L], *Me (IQR)* | 17.9 (4.85) | 16.5 (15.15) | 15.8 (2.4) | 0.064 |
| E/I ratio, *Me (IQR)* | 0.815 (0.14) | 0.74 (0.25) | 0.82 (0.2) | 0.219 |
| BCM [kg], *Me (IQR)* | 21.1 (8.975) | 18.95 (5.53) | 17.9 (5.7) | 0.199 |
| **Laboratory measurements** | | | | |
| Neut [G/l], *Me (IQR)* | 3.83 (1.81) | 3.49 (5.975) | 4.14 (6.83) | 0.556 |
| Mono [G/l], *Me (IQR)* | 0.565 (0.29) | 0.44 (0.795) | 0.5 (0.26) | 0.920 |
| Eos [G/l], *Me (IQR)* | 0.12 (0.14) | 0.14 (0.1) | 0.09 (0.15) | 0.374 |
| Baso [G/l], *Me (IQR)* | 0.02 (0.02) | 0.02 (0.01) | 0.02 (0.01) | 0.495 |
| Prealbumin [mg/dl], *Me (IQR)* | 26.7 (17.17) | 23.2 (24.65) | 29.75 (14.8) | 0.902 |
| Vitamin B1 [pM/l], *Me (IQR)* | 110.08 (200.28) | 48.63 (156.04) | 87.655 (96.58) | 0.181 |

Me- median; IQR – interquartile range; AntiScl70-Ab – anti -topoisomerase I autoantibody; AntiACA-Ab – anticentromere autoantibody; AntiRNAPIII-Ab – anti-RNA polymerase III autoantibody; OH - overhydration ; V – volume; TBW – total body water; ECW - extracellular water; ICW - intracellular water; E/I ratio – extracellular-to-intracellular fluid volume ratio; BMI – body mass index; BCM – body cell mass; HGS – handgrip strength; WC – waist circumference; WHR – waist-hip ratio; TSF – triceps skinfold thickness; Neut – neutrophils; Mono – monocytes; Eos – eosinophils; Baso – basophils.

**Table S2.** Comparison in GI symptoms at baseline between well-nourished, pre-cachectic, and malnourished SSc patients. Data are presented as n - numbers; % - percentage.

| | Well-nourished (n = 40) | Pre-cachexia (n = 5) | Malnourished (n = 11) | pValue |
|---|---|---|---|---|
| Difficulty swallowing, *n (%)* | 9 (22,5) | 1 (20,0) | 6 (54,5) | 0,112 |
| Reflux symptoms, *n (%)* | 15 (37,5) | 1 (20,0) | 4 (36,4) | 0,903 |
| Wakes up at night and chokes, *n (%)* | 2 (5,0) | 1 (20,0) | 1 (9,1) | 0,321 |
| Retrosternal burning, *n (%)* | 15 (35,0) | 1 (20,0) | 4 (36,4) | 0,906 |
| Early satiety, *n (%)* | 6 (15,0) | 1 (20,0) | 4 (36,4) | 0,279 |
| Abdomen swelling or bloating, *n (%)* | 9 (22,5) | 2 (40,0) | 3 (27,3) | 0,690 |
| Nausea, *n (%)* | 3 (7,5) | 1 (20,0) | 2 (18,2) | 0,248 |
| Constipation, *n (%)* | 8 (20,0) | 1 (20,0) | 4 (36,4) | 0,469 |
| Diarrhea, *n (%)* | 4 (10,0) | 1 (20,0) | 1 (9,1) | 0,777 |
| Took antibiotics for diarrhea, *n (%)* | 0 | 0 | 1 (9,1) | 0,291 |
| Steatorrhea, *n (%)* | 1 (2,5) | 1 (20,0) | 0 | 0,208 |
| Fecal incontinence, *n (%)* | 0 | 0 | 2 (18,2) | 0,078 |
| Requires parenteral nutrition, *n (%)* | 0 | 0 | 1 (9,1) | 0,286 |

**Table S3.** Comparison in treatment at baseline between well-nourished, pre-cachectic, and malnourished SSc patients. Data are presented as n - numbers; % - percentage.

| | Well-nourished (n = 40) | Pre-cachexia (n = 5) | Malnourished (n = 11) | pValue |
|---|---|---|---|---|
| azathioprine, n (%) | 5 (12,5) | 0 | 0 | 0,737 |
| steroids, n (%) | 10 (25,0) | 1 (20,0) | 5 (45,5) | 0,438 |
| mycophenolate mofetil, n (%) | 5 (12,5) | 0 | 1 (9,1) | 1,000 |
| methotrexate, n (%) | 2 (5,0) | 0 | 0 | 1,000 |
| cyclophosphamide, n (%) | 2 (5,0) | 0 | 0 | 1,000 |
| symptomatic drugs for Raynaud's phenomenon, n (%) | 36 (90,0) | 4 (80,0) | 10 (90,9) | 0,777 |

**Table S4.** The comparison between baseline (visit 1), 3 months after high protein oral nutritional supplement (visit 2), and 12 months (visit 3) since baseline in SSc patients with pre-cachexia. Data are presented as Me – median; IQR – interquartile range.

| | Visit 1 | Visit 2 | Visit 3 | P value |
|---|---|---|---|---|
| **Anthropometric measurements** | | | | |
| BMI [kg/m²], *Me (IQR)* | 24.8 (11.2) | 26.9 (10.1) | 26 (9) | 0.549 |
| HGS [kg], *Me (IQR)* | 16.7 (14.8) | 18 (14.8) | 17.6 (11.2) | 0.247 |
| WC [cm], *Me (IQR)* | 84 (17.5) | 84 (19.8) | 87 (18) | 0.211 |
| WHR [ratio], *Me (IQR)* | 0.9 (0.1) | 0.9 (0.1) | 0.9 (0.2) | 0.179 |
| TSF [mm], *Me (IQR)* | 21.1 (13.1) | 24.3 (15.6) | 21 (14) | 0.449 |
| **Bioimpedance analysis** | | | | |
| OH [L], *Me (IQR)* | -1.5 (7) | 0.8 (2.2) | 0.1 (2.6) | 0.074 |
| OH [% ECW], *Me (IQR)* | -11.6 (43.6) | 4.4 (13.1) | 0.4 (16.6) | 0.074 |
| V [L], *Me (IQR)* | 29.2 (20) | 31.1 (7.9) | 32.9 (6.8) | 0.819 |
| TBW [L], *Me (IQR)* | 30.6 (20) | 32.6 (8.1) | 33.1 (30) | 0.247 |
| E/I ratio, *Me (IQR)* | 0.7 (0.3) | 0.9 (0.2) | 0.9 (0.3) | 0.692 |
| Fat [kg], *Me (IQR)* | 17.6 (15.3) | 24.5 (22.8) | 20.5 (23.7) | 0.472 |
| Fat [%], *Me (IQR)* | 31.1 (10.5) | 30.2 (24.8) | 34.6 (23.5) | 0.779 |
| ATM [kg], *Me (IQR)* | 24 (20.8) | 33.3 (31) | 27.9 (32.2) | 0.472 |

| | | | | |
|---|---|---|---|---|
| **Laboratory measurements** | | | | |
| Neut [G/l], *Me (IQR)* | 3.5 (6) | 4.5 (4.1) | 3.5 (4.6) | 0.074 |
| Mono [G/l], *Me (IQR)* | 0.4 (0.8) | 0.6 (0.8) | 0.6 (0.8) | 0.091 |
| Eos [G/l], *Me (IQR)* | 0.1 (0.1) | 0.1 (0) | 0.1 (0) | **0.040** |
| Baso [G/l], *Me (IQR)* | 0 (0) | 0 (0) | 0 (0) | 0.156 |
| TC [mg/dl], *Me (IQR)* | 162 (47) | 207 (66) | 174 (55.5) | 0.074 |
| TG [mg/dl], *Me (IQR)* | 113 (54) | 119 (37) | 102 (70.5) | 0.449 |
| HDL [mg/dl], *Me (IQR)* | 45 (23.5) | 47 (27) | 43 (15.5) | 0.223 |
| LDL [mg/dl], *Me (IQR)* | 98 (36) | 127 (50.5) | 107 (52.5) | 0.074 |
| Prealbumin [mg/dl], *Me (IQR)* | 23.2 (24.7) | 24.5 (11.3) | 27.8 (10.5) | 0.819 |
| Vitamin B1 [pM/l], *Me (IQR)* | 48.6 (156) | 89.2 (158) | 22.3 (48.6) | 0.247 |

Me – median; IQR – interquartile range; OH - overhydration ; V – volume; TBW – total body water; E/I ratio – extracellular-to-intracellular fluid volume ratio; BMI – body mass index; FAT – fat mass; ATM – adipose tissue mass; HGS – handgrip strength; WC – waist circumference; WHR – waist-hip ratio; TSF – triceps skinfold thickness; Neut – neutrophils; Mono – monocytes; Eos – eosinophils; Baso – basophils; TC - total cholesterol; TG – triglycerides; HDL - high-density lipoprotein cholesterol; LDL - low-density lipoprotein cholesterol.

**Table S5.** The comparison between baseline (visit 1), 3 months after high protein oral nutritional supplement (visit 2), and 12 months (visit 3) since baseline in SSc patients with malnutrition. Data are presented as Me – median; IQR – interquartile range.

| | Visit 1 | Visit 2 | Visit 3 | P value |
|---|---|---|---|---|
| **Anthropometric measurements** | | | | |
| BMI [kg/m²], *Me (IQR)* | 20.7 (4.2) | 20.3 (5.2) | 21.1 (5.4) | 0.289 |
| HGS [kg], *Me (IQR)* | 18 (10.3) | 19.8 (6.3) | 25 (11.7) | 0.311 |
| WC [cm], *Me (IQR)* | 79 (11) | 79 (11) | 80 (10) | 0.651 |
| WHR [ratio], *Me (IQR)* | 0.8 (0.1) | 0.8 (0.1) | 0.8 (0) | 0.154 |
| TSF [mm], *Me (IQR)* | 15.9 (9) | 14.6 (9.1) | 13.5 (10) | 0.867 |
| **Bioimpedance analysis** | | | | |
| OH [L], *Me (IQR)* | 0.5 (1.8) | -0.3 (2) | 0.8 (1.3) | 0.156 |
| OH [% ECW], *Me (IQR)* | 3.2 (14.3) | -2.1 (14.5) | 5.7 (8.5) | 0.156 |
| V [L], *Me (IQR)* | 27.8 (3.5) | 29.8 (3.9) | 31 (3) | 0.054 |
| TBW [L], *Me (IQR)* | 28.8 (6.8) | 29.8 (4.5) | 31.9 (2.4) | 0.565 |
| E/I ratio, *Me (IQR)* | 0.8 (0.2) | 0.8 (0.1) | 0.8 (0.2) | 0.223 |
| Fat [kg], *Me (IQR)* | 18.3 (11.7) | 11.8 (9.7) | 17 (17.7) | 0.867 |
| Fat [%], *Me (IQR)* | 31.8 (20.2) | 22.7 (19.1) | 28.3 (25.3) | 0.368 |
| ATM [kg], *Me (IQR)* | 24.8 (15.9) | 16.1 (13.3) | 23.1 (24.2) | 0.867 |
| **Laboratory measurements** | | | | |
| Neut [G/l], *Me (IQR)* | 4.1 (6.8) | 4.4 (6.5) | 3.3 (1.4) | 0.607 |
| Mono [G/l], *Me (IQR)* | 0.5 (0.3) | 0.7 (1) | 0.5 (1.1) | 0.846 |
| Eos [G/l], *Me (IQR)* | 0.1 (0.2) | 0.2 (0.2) | 0.2 (0.3) | 0.128 |
| Baso [G/l], *Me (IQR)* | 0 (0) | 0 (0.1) | 0 (0.1) | 0.055 |
| TC [mg/dl], *Me (IQR)* | 241 (59) | 225 (33) | 245 (49) | 0.867 |
| TG [mg/dl], *Me (IQR)* | 196 (115) | 137 (119) | 129 (193) | 0.772 |
| HDL [mg/dl], *Me (IQR)* | 47 (27) | 56 (24) | 63 (24) | 0.651 |
| LDL [mg/dl], *Me (IQR)* | 153 (46) | 139 (28) | 153 (47) | 0.565 |
| Prealbumin [mg/dl], *Me (IQR)* | 34.7 (12.8) | 24 (16) | 31.2 (5.8) | 0.102 |
| Vitamin B1 [pM/l], *Me (IQR)* | 114.9 (87.8) | 104.8 (69.8) | 33.5 (109) | 0.180 |

Me-median; IQR – interquartile range; OH - overhydration; V – volume; TBW – total body water; E/I ratio – extracellular-to-intracellular fluid volume ratio; BMI – body mass index; FAT – fat mass; ATM – adipose tissue mass; HGS – handgrip strength; HC – hip circumference; WC – waist circumference; WHR – waist-hip ratio; TSF – triceps skinfold thickness; Neut – neutrophils; Mono – monocytes; Eos – eosinophils; Baso – basophils; TC - total cholesterol; TG – triglycerides; HDL - high-density lipoprotein cholesterol; LDL - low-density lipoprotein cholesterol.
